# Fear not: recent advances in understanding the neural basis of fear memories and implications for treatment development

**DOI:** 10.12688/f1000research.20053.1

**Published:** 2019-11-21

**Authors:** Amy L. Milton

**Affiliations:** 1 Department of Psychology, University of Cambridge, Cambridge, CB2 3EB, UK

**Keywords:** fear, amygdala, hippocampus, prelimbic, infralimbic, memory, reconsolidation, PTSD

## Abstract

Fear is a highly adaptive emotion that has evolved to promote survival and reproductive fitness. However, maladaptive expression of fear can lead to debilitating stressor-related and anxiety disorders such as post-traumatic stress disorder. Although the neural basis of fear has been extensively researched for several decades, recent technological advances in pharmacogenetics and optogenetics have allowed greater resolution in understanding the neural circuits that underlie fear. Alongside conceptual advances in the understanding of fear memory, this increased knowledge has clarified mechanisms for some currently available therapies for post-traumatic stress disorder and has identified new potential treatment targets.

## Introduction

Fear is a highly adaptive set of behavioural responses that have evolved to allow us to survive and reproduce. As an emotion, fear can be separated into three coordinated domains of response, including behavioural and physiological changes in addition to the subjective ‘feelings’ that are more colloquially referred to when considering emotional states. The basic emotional states of happiness, anger, fear, sadness and disgust are highly conserved across mammals (and arguably across the animal kingdom) as they confer strong evolutionary advantage. Therefore, the study of fear (and emotions more generally) is not restricted to humans or psychology and indeed has benefited markedly from research conducted in animal experimental systems. This has been especially pronounced in the field of behavioural neuroscience, where our understanding of the neural basis of fear has progressed markedly over the past 30 to 40 years (see 1 for review). In addition to the intellectual value of understanding the fundamental neural mechanisms of fear, there has been great clinical interest in understanding these mechanisms. A number of highly distressing and costly mental health disorders, including phobia and post-traumatic stress disorder (PTSD), have been conceptualised as having their roots in fear learning and memory ^2, 3^. Consequently, an understanding of how fear is instantiated in the brain may provide new avenues to treatment for these mental health disorders.

The study of fear in psychology and neuroscience has been primarily the study of fear learning and memory. Although innate fears do exist (that is, environmental cues can provoke a fearful response in the absence of any prior exposure, such as specific predator scents in rodents), the study of learned fears allows greater experimental control and provides insight into the neural mechanisms underlying learning and memory. Not only does this approach allow comparison of behaviour and neuronal responses before and after fear is learned, it also allows comparison with naïve control groups or, as is often the case in human studies, comparison with other ‘non-fearful’ cues in discriminative fear learning procedures. Learned fear is also relevant to mental health disorders – mostly obviously PTSD, which by definition develops following a traumatic and fearful experience ^4^ – providing translational potential. Furthermore, because fear learning can be pavlovian (and therefore under the control of the experimenter) and because robust fear learning can occur with a single learning trial, the study of fear memory has been extremely influential in studying memory processes such as memory consolidation ^5^ and reconsolidation ^6^. (See Table 1 for a definition of relevant psychological terms.) A further advantage is the possibility of ‘extinguishing’ fear through repeated cue exposure, so providing insight into how fear can be controlled. Indeed, fear extinction forms the basis for current PTSD and phobia therapies such as systematic desensitisation and prolonged exposure therapy, and so understanding the mechanisms underlying fear extinction provides insight into how these therapies work.

**Table 1.** Definitions of relevant psychological terms.

Term	Definition
B oundary conditions	This term refers to limits on the occurrence of the memory reconsolidation process. These have been hypothesised to include memory strength and memory age. These boundary conditions are usually defined on the basis of observations of a lack of amnesia (preserved memory) following the administration of an amnestic agent in conjunction with a specific memory reactivation procedure. The mechanistic underpinnings of these boundary conditions warrant further investigation, although a current view is that these can be related to synaptic metaplasticity mechanisms ^20^.
D estabilisation	The process by which a previously consolidated memory becomes unstable (labile) and enters a state in which it can be modified with new information.
E ngram	The representation of a memory within the brain, reflected by structural changes; a memory trace.
E xplicit	A subtype of long-term memory in which the content of the memory is consciously known.
E xtinction	This term can refer to the procedure of ‘extinction’, during which an animal or human is exposed to a previous learning situation but without reinforcement. This article refers predominantly to pavlovian extinction, where a previously reinforced conditioned stimulus is presented in the absence of the unconditioned stimulus. The term can also refer to the process of ‘extinction’, which has been hypothesised to require the formation of a new inhibitory memory trace but may also reflect some degree of unlearning (see ‘Insights into fear learning and memory’ section for details).
I mplicit	A subtype of long-term memory in which the content of the memory may not be consciously known (for example, emotional or procedural memories).
L earning	The process of acquiring information that will lead to a persistent change in behaviour following an experience.
M emory	The storage of learned information that leads to a persistent change in behaviour following an experience.
M emory consolidation	The process by which a memory is initially converted into a long-lasting trace; widely thought to be associated with changes in synaptic plasticity and engram formation.
M emory reconsolidation	The process by which a previously consolidated memory becomes destabilised and enters a state in which it can be updated and subsequently becomes re-stabilised through mechanisms thought to be partially overlapping with those engaged by memory consolidation.
O ccasion setter	A stimulus that is differentially associated with a particular reinforcement contingency and helps to resolve ambiguity over whether a stimulus will be reinforced.
P rediction error	A formal term for the mismatch between what is expected by an organism on the basis of previous experience and what actually occurs. This term is reflected in many models of learning and can be considered a signal that an engram requires updating. Reward prediction error has been particularly associated with dopaminergic signalling.
R eactivation	Outside of reconsolidation research, memory ‘reactivation’ is often used almost interchangeably with memory ‘retrieval’; but within the reconsolidation field, the term is used more specifically to refer to the procedure used to induce a previously consolidated memory to become unstable (that is, destabilised) or to the induction of the destabilisation process itself.
R etrieval-extinction	This is a procedure, first described by Monfils and colleagues ^21^, in which a reminder of previous learning, followed by a short delay and then extinction training, leads to a stronger and more persistent reduction in responding than extinction training alone. There is not yet consensus as to whether retrieval-extinction depends upon reconsolidation mechanisms and reflects updating of the original memory or whether it is a facilitation of extinction (see 22 for review).
T rauma film procedure	This is a procedure used to model intrusive memories akin to flashbacks in post-traumatic stress disorder (PTSD) in healthy experimental participants. Participants are shown film clips depicting events listed as qualifying traumatic events for PTSD in the *Diagnostic and Statistical Manual of Mental Disorders*, which induce intrusive memories of the film clips for several days following exposure.

With recent technological and theoretical advances, the study of fear memory has advanced markedly in the past few years. This review will consider the recent advances that have been made in understanding the neural circuitry underlying the expression of fear and extinction memories before considering the impact that this research may have on the development of novel treatments for disorders of fear learning.

## Insights into the neural circuitry of fear

**Figure 1.**
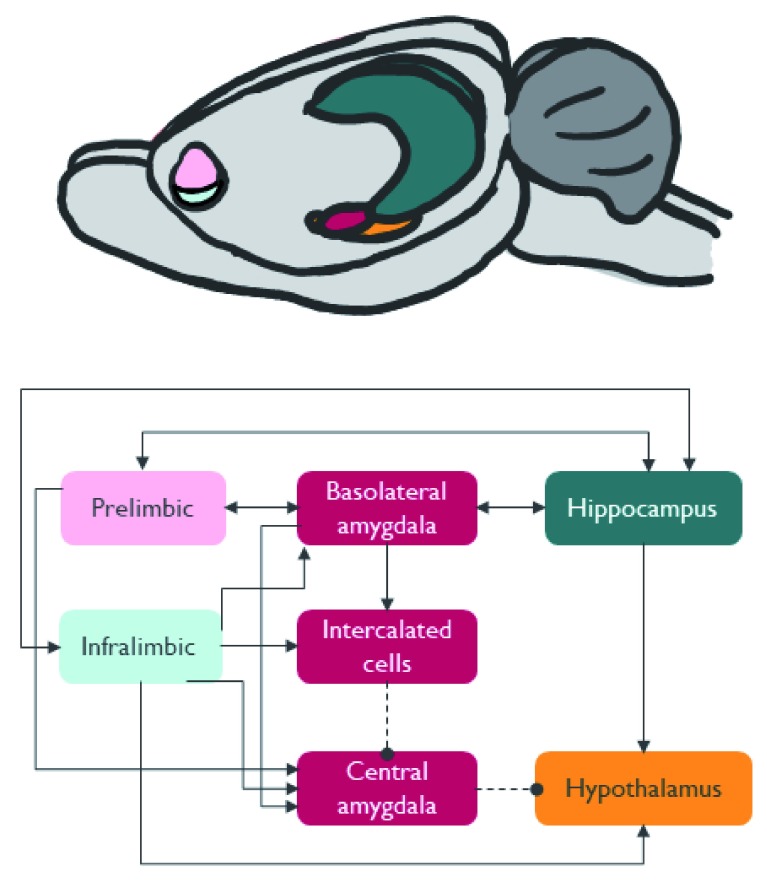
Illustration and schematic of key brain regions involved in fear and extinction learning. These structures form a highly interconnected network that produces the ultimate output of fear coordinated in the behavioural, neuroendocrine and autonomic domains. For a more detailed view of the subregions involved in fear learning and extinction, refer to Tovote and colleagues ^1^.

Fear is not produced by any single brain region but rather as the result of a network of brain structures that allow coordination of the behavioural, physiological and subjective response domains ( Figure 1; see 7 for review). It has been known for the past 30 to 40 years that a key brain region in supporting fear memory is the amygdala ^8^. This almond-shaped structure in the medial temporal lobe can be divided into multiple subnuclei, and the central amygdala (CeA) and basolateral amygdala (BLA) nuclei are critical for pavlovian fear conditioning. These amygdala nuclei interact with structures such as the hypothalamus and periaqueductal grey (PAG) nucleus in the brainstem to support autonomic and neuroendocrine responses to fearful cues, in addition to stereotyped reflexive responses such as conditioned freezing (the cessation of all movements except for those necessary for respiration) or aggressive responses, depending on the proximity of the threatening cue ^9^. Another region of critical importance, particularly with respect to encoding the timing of aversive outcomes with fearful cues, is the cerebellum (see 10, 11 for review). These circuits were originally determined through the use of lesion and inactivation techniques, but technological developments in neuroscience, such as pharmacogenetics – for example, designer receptors exclusively activated by designer drugs (DREADDs) ^12^ – and optogenetics ^13^, have provided insight into the subtleties of these structures’ contributions to fear behaviour. For example, it has been shown that the amygdala is capable of complex stimulus processing, including the coding of ambiguous aversive cues within the environment ^14^, and pharmacogenetic manipulation of the engrams underlying ambiguous cues induced a generalisation of fear memory while leaving the original fear memory intact ^15^. It has also been revealed that there are distinct projections from the CeA to the PAG that control active (for example, flight) and passive (for example, freezing) fear responses ^16^ and that these projections communicate with different neurochemical signals. Interestingly, converging evidence from human functional neuroimaging studies has shown recruitment of different parts of the PAG during active and passive fear responses elicited by an approaching ‘predator’ in a video game ^17^. Thus, far from being a simple ‘output nucleus’, the PAG contributes to the choice of appropriate fearful behaviour dependent on environmental conditions.

The role of the hypothalamus also appears to go beyond an ‘output nucleus’. It has long been known that the medial hypothalamus is required for the expression of defensive responses directed at predators ^18^. However, recent work using techniques to label neurons constituting a specific memory trace has shown that fear engrams are present within the ventromedial hypothalamus (VMH) and that selectively silencing these neurons prevents both the acquisition and subsequent recall of a fear memory ^19^. Furthermore, fear engrams in the paraventricular and supraoptic nuclei of the hypothalamus have been labelled, and the projection of these neurons to the CeA is critical for the expression of fearful behaviour ^23^. Thus, rather than reflecting simple ‘output’ structures of the amygdala, the brainstem and hypothalamic nuclei represent a more distributed circuit that can fine-tune the expression of fearful behaviour.

Studies of fear memory extinction have focused primarily on regions of the prefrontal cortex. It is important to note that extinction is widely considered to reflect inhibition of the original fear memory by a competing ‘cue-no fear’ memory rather than ‘unlearning’ of the original fear memory trace ^24^. Consistent with the requirement for prefrontal regions in the regulation of other cognitive processes, studies of extinction have focused on prefrontal regions as regulators of structures such as the amygdala. Lesion and electrophysiological studies conducted in the 1990s and early 2000s showed that the rodent prelimbic cortex is required for fear memory expression, and that infralimbic (IL) cortex is required for the extinction of fear and the expression of the extinction memory ^25– 27^. As for studies of the hypothalamic contribution to fear, understanding of the prefrontal contribution to fear has been deepened with the advent of pharmacogenetic and optogenetic techniques: for example, optogenetic stimulation of the IL cortex has revealed that conditioned freezing can be reduced independently of extinction learning ^28^. Furthermore, the functional coupling between the IL cortex and the amygdala can be modulated by sex hormones such as oestradiol in female rats, which increases the activity of the IL cortex during the retrieval of an extinction memory (that is, when fear is suppressed) ^29^. This mechanism appears to recruit learning mechanisms as it is dependent on the *N*-methyl- d-aspartate (NMDA) subtype of glutamate receptor ^30^ (which is required for memory storage) and interestingly appears to be dependent on female reproductive experience ^31^. These data provide further insight into the mechanisms underlying the well-documented sex differences in the prevalence of fear and anxiety disorders. Females are known to respond differently to fear learning and extinction procedures depending on circulating hormone levels ^32^ (but note that males also show a dependence on oestradiol for fear extinction ^33^) and to show differences in fear memory generalisation ^34^ that may contribute to the higher prevalence of fear and anxiety disorders in women.

Once the fear and extinction memory traces have been acquired, the brain must have a strategy to determine which trace should dominate behaviour under specific circumstances. A highly influential view suggests that external contexts (for example, configurations of environmental cues) and internal contexts (for example, interoceptive states) can act as occasion setters and arbitrators between the two engrams ^35^. This view further suggests that the original fear memory is more likely to generalise across time and space than the extinction memory, which is more context-specific. In this way, it is possible to account for Pavlov’s findings ^36^ of different psychological routes for the return of the original fear memory: spontaneous recovery (the return of fear following a change in internal states with time), reinstatement (the return of fear following a return to the fear-associated environment or re-exposure to the fear-eliciting stimulus) and renewal (a return of fear in environments other than the ‘safe’ extinction environment).

The hippocampus has been strongly implicated in the representation of space and contexts and consequently has received great research interest both for its role in contextual fear conditioning and for contributing to occasion setting during extinction. Different projections from the hippocampus to the amygdala appear to be important for these two processes. While ventral hippocampal projections to the basal amygdala support contextual fear memory – consistent with hippocampal lesion data ^37^ – projections to the CeA support the capacity of a context to act as an occasion setter for cued fear memory retrieval ^38^.

The requirement for the hippocampus, in contrast to the amygdala ^39^, in the storage of contextual fear memories appears to be time-limited ^40^. For recent memories, it has been shown that the same population of hippocampal neurons is activated during the encoding and the retrieval of a fear-associated context, as shown by measures of protein kinase expression ^41^, engram labelling ^42^ and calcium imaging ^43^. Increases in spine density – reflective of synaptic plasticity – have also been observed in the hippocampus following fear conditioning, although these appear to be time-limited, and remote memories are associated with spine density increases in the anterior cingulate cortex (ACC) ^44^. These changes in spine density appear to be sequential, as preventing spine density changes in the hippocampus precludes subsequent changes in the ACC ^44^. The time-limited nature of hippocampal plasticity in memory storage is also observed in studies of neurogenesis. Increasing hippocampal neurogenesis shortly after the acquisition of a fear memory leads to impaired retrieval of that memory, but increasing neurogenesis at later time points does not, most likely because the memory is no longer dependent on the hippocampus for its expression ^45^. Consistent with a time-limited requirement for the hippocampus in the retrieval of fear memories, human functional imaging studies have shown that the strength of hippocampal connectivity with neocortical regions increases following fear conditioning and that the strength of this connectivity correlates with fear behaviour at a retention test conducted 24 hours after learning ^46^.

Much of our understanding of the neural circuity underlying fear and anxiety disorders has been driven in a ‘bottom-up’ manner from fundamental neuroscience studies of fear learning in animals. Some recent advances, however, have derived from a ‘top-down’ approach that has attempted to provide a mechanism for therapies currently used to treat mental health disorders such as PTSD. One such therapy is eye movement desensitisation and reprocessing (EMDR) ^47^, which is recommended as the second line of treatment for PTSD by the UK National Institute for Health and Care Excellence (NICE). During EMDR, patients and therapists work together to identify disturbing thoughts, mental images and feelings that become potential targets for ‘reprocessing’ through extinction. A psychological ‘safe place’ is identified by the patient and used to help tolerate stress elicited by trigger cue re-exposure during the therapy. In the therapy itself, the patient is guided through the disturbing thoughts by the therapist while performing a visuospatial task such as visually tracking a moving target. This ‘reprocessing’ occurs until the patient reports loss of fear to the trigger stimuli. Although EMDR has been praised in the clinical literature for its efficacy ^48^, the mechanism by which EMDR reduces fear has been a matter of debate ^49– 51^. Though it has been established that a visuospatial task performed concurrently with fear memory extinction leads to deactivation of the amygdala ^52^, there remains debate as to whether the efficacy of EMDR depends critically upon the visuospatial component of the therapy or whether this is simply an epiphenomenon.

Recent research has supported the importance of the visuospatial manipulation in EMDR and identified a neural mechanism by which repetitive, side-alternating eye movements could lead to long-term reductions in fear. The superior colliculus of the midbrain is critically important for eye movements and has been shown to mediate the effects of eye movement on amygdala activity. With a version of EMDR backtranslated for mice, it was shown that alternating bilateral sensory stimulation led to enhanced activity in ‘extinction’ memory traces within the amygdala and that this enhanced activity depended on a circuit involving projections to the amygdala from the superior colliculus via the mediodorsal thalamus ^53^. The superior colliculus has been further implicated in mediating defensive behaviours to fearful cues ^54^, although it remains to be determined whether this recruits the same circuitry as the backtranslated EMDR procedure.

## Insights into fear learning and memory

As noted above, the robust and rapidly learned nature of fear memory makes it an ideal model system for investigating memory processes such as learning (memory acquisition), storage (consolidation) and updating and persistence (reconsolidation). Indeed, the traction gained by research into memory reconsolidation, which had originally been described in the 1960s ^55, 56^ but was rediscovered at the turn of the century ^57^, can be attributed in large part to the use of the psychologically and neurobiologically well-characterised procedure of pavlovian fear conditioning, as well as the use of amnestic agents with more readily defined mechanisms of action, such as protein synthesis inhibitors. In addition to the utility of fear for studying the fundamental properties of memory, views of disorders such as specific phobia and PTSD emphasise the importance of learned cue-fear associations in the development and persistence of these disorders ^58^. Therefore, the manipulation of pavlovian fear memories – whether disrupting cue-fear memories or enhancing the fear memory extinction – is a plausible therapeutic target for anxiety disorders ^59, 60^.

Much research into fear learning and memory has focused on the amygdala, as it has been strongly implicated in pavlovian fear learning and the persistence of fear memories. Several lines of evidence suggest that, unlike memories dependent on the hippocampus, cue-fear memories are permanently stored within the amygdala once acquired ^39, 61, 62^, although this is not universally accepted (see 63– 65 for review) and may depend on how the fear memory is tested (for example, active versus passive defensive responses). More recently, there has been interest in whether the amygdala also undergoes synaptic plasticity changes following fear memory extinction. Although traditionally extinction has not been conceptualised as ‘unlearning’ ^24^, since the identification of ‘fear’ and ‘extinction’ engrams in the amygdala ^66^ and in light of detailed structural studies showing that extinction is followed by elimination of dendritic spines formed during fear conditioning in the amygdala ^67^ and auditory cortex ^68^, there has been increasing interest in determining the degree to which synaptic plasticity is required in the amygdala for the expression of fear extinction. Our own research has shown that extinction engages key molecular markers of synaptic plasticity within the amygdala, including protein kinases such as extracellular signal-regulated kinase (ERK) ^69^ and the protein phosphatase calcineurin ^70^. Whereas ERK is also recruited for fear memory reconsolidation, calcineurin appears to be specific to the formation of the extinction memory, and knockdown of calcineurin prevents the retention of an extinction memory without affecting the reconsolidation of the original fear memory ^70^. However, a limitation of this approach is that it cannot determine whether the synaptic plasticity events are occurring in the same or a different population of neurons within the amygdala (although the latter is perhaps more likely given the previous demonstrations of separate fear and extinction engrams). Though there is an increase in the expression of the immediate early gene *arc* in the amygdala during both fear memory reconsolidation and the consolidation of a fear extinction memory, this appears to be in distinct amygdala circuits ^71^.

Recent work has indicated that there are at least two classes of amygdala neurons recruited in extinction. It was recently found that the neurons recruited for the ‘extinction trace’ are parvalbumin-positive interneurons ^72^ and that activation of these neurons directly suppresses the expression of the original fear memory. These are separate to a population of glutamatergic neurons expressing the antigen Thy-1, which are thought to overlap with the ‘extinction’ neurons originally described by Herry and colleagues ^66^. The activation of Thy-1 neurons, in contrast to the parvalbumin-positive neurons, appears not only to facilitate the consolidation of an extinction trace but also to impair the reconsolidation of the original fear memory ^73^. These different molecular mechanisms may also reflect a shift in how extinction is instantiated following different degrees of non-reinforced cue exposure. It has been shown that, in the early stages of (massed) extinction training, the activation of both the amygdala and the IL cortex increases but that this activation returns to baseline levels with further (spaced) extinction training ^74^. It has been argued that these changes in neural recruitment may reflect the initial formation of an inhibitory extinction trace, followed by erasure of the original fear memory. This is consistent with other studies using spaced extinction training, in which a hippocampal fear memory engram was observed to be reactivated to incorporate new information during extinction training ^42^. However, in the latter study, the original engram persisted in its updated form. Whether this reflects differences in the storage of fear and extinction memory engrams in the hippocampus, amygdala and prefrontal cortex warrants further investigation.

## Treatments for fear and anxiety disorders: understanding mechanisms and identifying new approaches

In addition to informing scientific understanding of a fundamental memory process, studies of fear learning have the potential to identify how to optimise currently available treatments and to develop novel therapeutic approaches for fear and anxiety disorders. Whilst a major focus of recent work has been the study of neurochemical systems contributing to fear and anxiety, there has also been great interest in targeting the maladaptive fear memories that underlie fear and anxiety disorders.

Selective serotonin reuptake inhibitors (SSRIs) are widely used to treat anxiety and fear disorders such as PTSD ^75^, and recent research has aimed to clarify how these drugs exert their effects in the brain. Serotonin is released by the Raphé nucleus of the brainstem, and recent research has characterised in detail the circuits by which different Raphé nuclei contribute to fear and anxiety. It was recently shown that serotonin from the dorsal Raphé activates a population of corticotropin-releasing factor (CRF)-expressing neurons within the bed nucleus of the stria terminalis (BNST), which in turn activate a microcircuit within the BNST that regulates anxiolytic output on to the ventral tegmental area (VTA) and lateral hypothalamus ^76^. This circuitry appears to show sexually dimorphic baseline activity, and unstressed females show greater functional connectivity between regions such as the dorsal Raphé, amygdala and medial prefrontal cortices than unstressed males ^77^. Furthermore, CRF administration into the lateral ventricle led to differences in the limbic circuitry recruited in males and females, and there was greater co-activation of the BNST and nucleus accumbens, the PAG and nucleus accumbens, and the BNST and the septum and hippocampus in females than in males ^77^. This circuitry was modulated by oestradiol in unstressed but not stressed females, perhaps linking to the different prevalence of anxiety disorders in women, and sex differences in fear learning and extinction in rodents, as discussed above. In contrast to the dorsal Raphé, the median Raphé may be directly involved in the formation of fear memory, as optogenetic activation of the median Raphé in place of an electric shock was sufficient to generate a fear memory following a 7-day incubation period ^78^. Serotonin transporter (SERT) expression itself appears to be related to fear learning, as constitutive SERT ^-/-^ and SERT ^+/-^ rats show enhanced acquisition of fear learning and delayed extinction learning ^79^.

Another neurotransmitter that has been extensively investigated in fear research is dopamine. The relationship between serotonin and dopamine is still a matter of debate ^80^, and much of the evidence emphasises the importance of dopamine in appetitive learning and ‘reward’ prediction error ^81, 82^. However, increasing evidence indicates that dopamine can signal prediction error more generally and may contribute to the encoding of different types of prediction error in different brain structures ^83^. A population of dopaminergic neurons from the dorsal Raphé and ventral PAG are activated by the presentation of unpredicted electric footshocks, enhancing dopamine-dependent synaptic plasticity within the amygdala ^84^. These effects on synaptic plasticity likely reflect dopamine’s neuromodulatory function across a number of brain structures. Certainly, dopaminergic signalling has been shown to be necessary for the consolidation of fear memory in several structures involved in fear learning, including the hippocampus ^85^ and the amygdala ^86– 88^.

By contrast, and consistent with their role in reward prediction error, a dopaminergic population within the VTA is activated when predicted aversive outcomes do not occur and are necessary for the acquisition of extinction ^89^. However, a separate projection from the VTA to the medial prefrontal cortex appears to oppose extinction learning ^89^. The nigrostriatal dopamine system has also been implicated in fear extinction, and pharmacogenetic activation of this projection leads to enhanced retention of extinction and a reduction in context-induced renewal of fear ^89^. This correlated with the expression of c-Fos in the CeA and with activation of D _1_-expressing neurons in the dorsomedial striatum. As pharmacological activation of the D _1_ subtype of dopamine receptor only blocked fear renewal, this may suggest the recruitment of habitual strategies making the expression of the extinction memory less sensitive to the context.

Chronic pharmacological therapy is potentially not the only option for the treatment of anxiety and fear disorders. Since the turn of the century, there has been great interest in directly targeting the maladaptive fear memories that contribute to disorders such as PTSD. Although PTSD can potentially be treated by administering amnestic drugs immediately following the trauma ^90^, it is still advantageous to develop treatments that can be given even once the memory is well established. This would allow the treatment of PTSD in patients with remote trauma memories and would avoid ethical issues such as obtaining informed consent in acutely traumatised patients. One such approach is the disruption of memory reconsolidation, which has been extensively investigated with respect to pavlovian fear memories.

To disrupt reconsolidation requires two conditions: the destabilisation of the memory and the administration of an amnestic treatment. One of the most widely used pharmacological treatments to induce amnesia in reconsolidation procedures is the beta-blocker propranolol. This drug was one of the first drugs targeting a neurochemical system shown to disrupt fear memory reconsolidation in rodents ^91^, and it is approved for human use, facilitating translation to human studies. Propranolol, given in conjunction with memory reactivation, disrupts the reconsolidation of implicit (though not explicit) conditioned fear memories in healthy human volunteers ^92^ and reduces physiological responses to fear in patients with PTSD ^93– 95^ and phobia ^96^. Although there have been some failures to replicate these effects ^97^, this is likely due to difficulties in destabilising the target memory – so-called boundary conditions ^98^. Recent work has begun to investigate whether memories can be more effectively destabilised by re-exposure directly to the outcome rather than cues predictive of the outcome (that is, re-exposure to the unconditioned shock stimulus rather than conditioned stimulus) ^99^. It is difficult to envisage how this outcome-based reactivation, though of theoretical interest, could be translated to a therapeutic setting, although it is possible that re-exposure techniques based on virtual reality ^100^ could be adapted for this use.

Another approach to disrupting the reconsolidation of fear memories is the use of behavioural interference techniques rather than a pharmacological amnestic agent. This was first demonstrated through the use of extinction training shortly after the reactivation of the memory in rats ^6^ and humans ^101^ in what are now described as ‘ retrieval-extinction’ procedures. This retrieval-extinction approach is hypothesised to update the original fear memory with the safety (cue-no fear) memory rather than creating an alternative extinction memory that competes with and inhibits the original fear memory. Though potentially very impactful, the phenomenon has not been universally observed, and there have been failures to replicate in both rats ^102^ and humans ^103^. Furthermore, other studies have shown that the reduced recovery of fear associated with retrieval-extinction can be observed even when the mechanisms underlying memory destabilisation are blocked ^104^. As for propranolol, the interaction between potential boundary conditions and the mechanisms engaged warrants further investigation and likely corroboration with alternative forms of evidence, such as molecular analyses ^22^.

One final though relatively new approach aims to interfere with the reconsolidation of the cue representation that triggers intrusive thoughts and involuntary flashbacks in PTSD. It has been shown in healthy volunteers trained on the ‘ trauma film procedure’ ^105^ that it is possible to induce intrusive sensory memories reminiscent of flashbacks in PTSD and that the frequency of these intrusive memories can be reduced with the use of reconsolidation interference strategies. These highly visual sensory memories are hypothesised to depend on the same psychological and neurobiological substrates that are engaged by other highly engaging visuospatial tasks, such as the video game Tetris. Following demonstrations that Tetris played shortly after exposure to trauma films could reduce the consolidation of the visual sensory memories underlying flashbacks ^105^, it was shown that fully consolidated visual memories could also be disrupted by Tetris gameplay following a brief reactivation of the trauma film memory ^106^. This ‘behavioural interference’ approach has subsequently been shown to impair the consolidation of intrusive memories underlying flashbacks in clinical populations of road traffic accident survivors ^107^ and mothers who have undergone emergency caesarean sections ^108^. Furthermore, in a preregistered open-label study of patients with long-standing complex PTSD, visuospatial interference significantly reduced the number of intrusive thoughts of targeted trauma memories ^109^. These non-pharmacological methods to disrupt the reconsolidation of trauma memories warrant testing on a larger scale ^60^.

## Conclusions

Fear is a basic and highly adaptive emotion, but maladaptive fear can lead to debilitating mental health disorders. Recent advances in fear research have provided insight into the detailed neural circuits that support fearful behaviour and allow its modulation while also allowing a better understanding of currently available treatments for anxiety disorders. A deeper understanding of fear learning has also identified some potential novel treatment avenues that warrant further research in progressively more translational models ^110^.

## Abbreviations

ACC, anterior cingulate cortex; BNST, bed nucleus of the stria terminalis; CeA, central amygdala; CRF, corticotropin-releasing factor; EMDR, eye movement desensitisation and reprocessing; ERK, extracellular signal-regulated kinase; IL, infralimbic; PAG, periaqueductal grey region; PTSD, post-traumatic stress disorder; SERT, serotonin transporter; VTA, ventral tegmental area 
